# Can Social Cognitive Theory Influence Breakfast Frequency in an Institutional Context: A Qualitative Study

**DOI:** 10.3390/ijerph182111270

**Published:** 2021-10-27

**Authors:** Jessica A Harris, Julia Carins, Sharyn Rundle-Thiele

**Affiliations:** Social Marketing at Griffith, Griffith Business School, Griffith University, 170 Kessels Road, Nathan, QLD 4111, Australia; j.carins@griffith.edu.au (J.C.); s.rundle-thiele@griffith.edu.au (S.R.-T.)

**Keywords:** breakfast consumption, social cognitive theory, institutional feeding

## Abstract

Breakfast is considered an important meal, especially for people who are about to commence a long or demanding workday, and for roles that may involve physical tasks and a requirement to remain alert and vigilant in potentially high-risk situations. This study looks at breakfast consumption influences within two workplace institutional settings, namely military and mining. Semi-structured interviews were conducted with military personnel (n = 12) and mining employees (n = 12) to understand their breakfast consumption behaviour at work and at home, and the associated behavioural influences. The interview questions were framed by social cognitive theory. Overall, cognitive and environmental influences were the most prominent influences on breakfast consumption, less evident were behavioural influences. A negative stereotype of workplace institutional food services emerged as one of the most significant barriers to breakfast consumption for those already at work. Considerations of environmental influences on behaviour may need to be broadened beyond physical barriers and social influences, to include perceptions of the behavioural environment. Programs that aim to increase breakfast consumption must create areas where their employees want to go. Food systems need to ensure nutritious, quality, and appealing food is available. Interventions need to increase participants’ knowledge, improve their attitudes, and create positive expectations for breakfast.

## 1. Introduction

Breakfast is considered to be an important meal within the context of daily food intake [1]. This is especially so for people who are about to commence a long or demanding workday that may involve physical tasks and a requirement to remain alert and vigilant in potentially high-risk situations. Some of these occupations (for example, the military and mining) require personnel to live away from home, and meals are provided by institutional catering services. While this may appear convenient, it may also interfere with regular or preferred breakfast eating patterns. Research indicates that many military personnel and mining employees (or fly-in fly-out workers) do not follow healthy dietary habits [2,3], but the impact of this lifestyle on breakfast eating is mainly unknown. This study seeks to understand the influences on breakfast eating in this context to inform strategies to assist the delivery of increases in healthy breakfast consumption for personnel working away from home.

### 1.1. Breakfast Eating

Eating breakfast is considered to be a healthy behaviour, with breakfast skipping linked to non-communicable diseases, overweight, and obesity [4,5,6,7]. Regular breakfast skipping can also be related to other unhealthy behaviours, such as consuming a poorer diet and low physical activity, which also contribute to higher body mass index (BMI) [8,9]. On the other hand, breakfast diets rich in high quality protein have shown to be effective in appetite control and satiety [10,11]. Eating breakfast also supports cognitive performance, which is paramount for both physical and mental work. Studies have shown that skipping breakfast interferes with learning and has adverse reactions for memory [12,13]. While breakfast consumption has been studied in home or school breakfast settings [14,15,16,17,18], breakfast consumption in workplace institutional settings for adult populations has not been widely explored [19,20,21,22]. These workplace institutional settings cater to people who work in these areas; however, food options, timings, and locations can constrain choice and affect dietary behaviours.

### 1.2. Workplace Institutional Feeding

Institutional feeding provides meals to various consumers within Australia from health care settings, prisons, military, and workplace settings. In Western countries, 10–15% of foodservice meals are provided in institutional settings [22]. This involves the production and processing of foods from breakfast to dinner for the community across a variety of institutions. There is growing recognition of the importance of providing good quality and nutrient-rich food in institutional settings [23,24], and also an understanding that dietary preferences and needs vary and include vegetarians, vegans, and a wide range of other nutritional conditions, such as food allergies [25].

Many workplace institutions operate canteen or buffet style services, for example, those found in military dining and fly-in fly-out workplace settings (namely mines). These meals are self-service from bain-maries, allowing the individual to choose portion size and food options [22]. In most institutional food services, meals are offered based on a rotating or cyclical menu offering many alternatives. In many of these settings, the individual relies on the institution to provide most of their food throughout the day. Some workplace institutional populations work long or demanding days, involving physical tasks or a requirement to remain alert and vigilant in potentially high-risk situations. For these groups, it is essential to understand whether institutional feeding supports them to perform healthy behaviours (like breakfast eating) and how this imposed setting interacts with individual behavioural influences.

### 1.3. Australian Military Personnel

Military personnel need to be physically and mentally fit to serve the country and to be military ready at all times [26]. As all soldiers go through training to be “warfit” and to “build and sustain physical and mental resilience” [26], overall health is an integral part of ensuring peak performance. The prevalence of obesity in the military has also been examined by several researchers who have found that military populations have high obesity rates globally, similar to civilian population rates [26,27,28,29,30]. In a US survey, 10% reported needing to lose weight before joining the military, and across all ages, 51% were overweight [31]. The behavioural influences that lead to obesity in the military have been observed to be similar to those of the civilian population, such as social norming, lack of access to healthy foods, and peer group behaviour [28]. Research undertaken in the United States [31] found that many military personnel do not eat a healthy diet, with only 38.7% meeting daily fruit requirements and 22.2% meeting vegetable requirements [32]. Australian studies also suggest that military recruits are at risk of not meeting their daily nutritional needs, with evidence showing they consume a diet that is unsuitable for vigorous activity [3].

Furthermore, data from a major ADF training establishment found over 60% of personnel reported not eating breakfast daily [33]. This is concerning given that studies indicate that a balanced and optimal diet (including breakfast) is fundamentally important for military readiness, physical performance, and recovery [34,35,36]. Researchers have examined how to increase healthy eating within the military [34,35,37,38,39,40]; however, none of these have looked at one particular eating behaviour in isolation, nor have they looked at breakfast eating. Although the consequences of breakfast skipping have been examined in the general population [26,27,28,29,30], breakfast consumption in workplace institutional settings and the potential impacts on cognitive and physical performance have been overlooked.

### 1.4. Australian Mine Employees

Like military personnel, miners need to be in optimum physical and mental health to perform their job. Miners often work 12-h shifts, sometimes longer, which is both physically and cognitively draining. In a recent study completed on Australian miners, over 52% of the employees within the survey experienced stress and were contemplating stress management actions [41]. Workplace stress results in “employee tardiness, absenteeism, low productivity, high employee turnover, wasted investment in training, increased costs due to training replacements for sick leave, depression, aggression, and violence” [42,43]. Workplace stress has also been linked to unhealthy behaviours such as inadequate diet [41,44]. The Australian mining industry has the highest proportion of overweight and obese employees within any Australian organisation, with 76% overweight or obese measured by body mass index [2]. Being overweight results in productivity impairments and mobility issues for certain roles [45]. Despite high obesity rates, research has shown mining employees did not feel the need to improve their nutrition, and employees who were not contemplating change were less likely to ask for help. Hence, there is a clear need to improve healthy behaviours in the mining industry [2]. With little empirical evidence on breakfast eating behaviours within the Australian mining industry context, this research responds to calls to further support workplace health and wellbeing [41,46,47].

### 1.5. Social Cognitive Theory

Previous interventions that aim to improve breakfast consumption have been dominated by educational approaches [48,49,50,51,52]. Most interventions fail to report the use of theory, or do not explain, in detail, how the application of theory underpinned intervention design [51,53,54,55]. However, eating behaviours are influenced by more than cognitive elements (such as knowledge), they are also shaped by influences surrounding the individual [17,56]. Social cognitive theory (SCT) recognises the reciprocal determinism of behaviour, where both the person influences their surroundings and the surroundings influence the person [57].

SCT involves three triads of human behaviour: individual cognitive factors of knowledge, expectations, and attitudes; behavioural factors of skills, practice, and self-efficacy; and environmental factors of social norms, access, and influence of others on their environment [58,59], with all three factors working together to drive behaviour. Other studies that have used this model in healthy eating studies only use part of the model, selecting one or more of the factors, but never using all nine factors. Self-efficacy, practice, expectations, access, and social norms were undertaken by [60], while Brimblecombe, Ferguson [56] used self-efficacy, practice, knowledge, access, and influence, and Foley, Shrewsbury [61] measured self-efficacy, knowledge, attitude, and social norms. From these healthy eating studies, they all measured healthy eating through self-efficacy and then a number of other factors in cognitive and environmental triads. This paper used all nine factors within each triad to ensure the full theory was used in order to explore breakfast eating behaviours.

Theories (such as SCT) assist researchers and practitioners in understanding the links and influences on specific behaviours, ensuring that understanding is extended beyond how individuals think and feel, and are considered to support more effective intervention design and evaluation [51,55]. This study explores breakfast consumption within two institutional feeding settings using a SCT lens to provide foundational understanding to guide future intervention design aiming to improve breakfast eating rates. Given that not much is known about breakfast consumption in workplace institutional settings, this study used qualitative research to understand what personnel/workers experience and to begin to map the influences on behaviour.

This study aimed to identify how different SCT factors (personal, behavioural, and environmental) influenced breakfast consumption in workplace institutional settings. Interviews were used to provide rich insights into participants’ breakfast behaviours and explore their thoughts and experiences with breakfast consumption in these institutions.

## 2. Materials and Methods

Qualitative research helps to unpack the issues and explore how they are understood by those involved. This research aimed to explore breakfast consumption (as a healthy nutrition behaviour) within a setting likely to include a reciprocal relationship between the individual and the environment. For this purpose, the researchers selected two away-from-home settings that provided workplace institutional feeding services for employees, namely the Australian military and Australian mining sites. This study used one-on-one interviews to gain insight into breakfast eating, whether at home or when accessing institutional feeding. The discussion of breakfast eating in both home and work contexts was used to prompt a discussion of the many and varied environmental, behavioural, and personal factors that influence breakfast eating.

### 2.1. Participation and Recruitment

The study aimed to conduct a total of 24 interviews, with 12 participants from each workforce. Twelve interviews are considered sufficient to reach data (or coding) saturation when using semi-structured formats with relatively homogenous groups [62]. Participant recruitment employed a convenience sampling approach, using broadcast emails through organisational contacts and social media advertising, both of which invited participants to register their interest through a web link. Each participant was then sent an introductory email with information about the study requesting 30 min of their time for a one-to-one interview. All interviews were kept anonymous; researchers and interviewees were not known to each other, and no further contact between the researchers and participants outside the interview were conducted. Two researchers from the authorship team contributed to the selection and refinement of interview questions to ensure they reflected the theoretical framework and to reduce the likelihood of personal bias shaping the questions. The analysis and interpretation of the data were completed by two independent researchers separately and then discussed to resolve any differences to minimize the impact of any researcher biases occurring and potential human errors when coding or omitting data. Researchers declared their own interests in the research and provided information on the funding body (Defence Scientific Technology Group and Griffith University) to each participant both verbally and through the signed consent forms, including information that the research formed part of a PhD program for one researcher. It was expected that personnel would have many job-related time commitments, so the interviews were designed to take 30 min. A 30-min semi-structured one-on-one interview can pragmatically be enough time to succinctly get the information required [63]. Participants were given consent forms, and all personal details were kept anonymous. Within the consent forms, participants were notified that at the end of the project that aggregated findings would be reported to funders and in the scientific literature. Participants were provided with the researchers contact details and invited to contact the researchers if they wanted copies of the study findings. After the completion of informed consent procedures, phone interviews were conducted due to COVID-19 restrictions at the time. The study was approved by Griffith University Human Research Ethics Committee and the Defence Science and Technology Group Low-Risk Ethics Panel (protocol numbers 2019/382 and LD-12-20).

### 2.2. Procedure

The interviews explored breakfast eating, focussing on breakfast eating experiences over the previous seven days. The aim was to understand firstly their current breakfast eating patterns and then their previous patterns during earlier life stages to gain insights into any potential habit transitions or interruptions [4,64,65,66,67]. The primary theoretical constructs explored with the participants were cognitive aspects related to breakfast eating, including assumptions about breakfast, and expectations and attitudes towards breakfast eating; environmental influences including access, social norms, and social influence; and behavioural constructs including self-efficacy, skills, and practice. A series of open-ended questions were asked, allowing respondents to provide rich and unconstrained answers [68]. The interviews followed a semi-structured format, with examples of the questions used in the interviews as follows:

Behavioural: How has your breakfast eating changed from adolescence to now? What do you expect to happen after eating breakfast?

Personal: Tell me about the last breakfast you ate. Can you describe your first ever memory of eating breakfast? Can you describe a typical breakfast scenario for your teenage years?

Environmental: Does your worksite encourage you to eat breakfast? What do they usually say? When you are on the worksite, what kind of breakfast is provided each morning? How are the breakfasts provided on the worksite different from home?

A complete interview question list can be found in Appendix A.

Probing and reframing questions were used to encourage participants to provide detail in response to specific questions and assist clarification. All interviews were recorded to allow the moderator to focus on verbal communication and ensure attention could be given to subtle cues, and later the interviews were professionally transcribed to accurately capture spoken detail [69].

### 2.3. Analysis

Analysis of the interview transcripts followed a thematic approach [70] using NVivo as an organisational data tool to assist the researcher in understanding, organising, and interpreting the data. NVivo is a qualitative database used to process complex data. Each transcribed interview was inserted into NVivo for coding [71]. Thematic analysis was used to categorise, examine, and report themes consistent with SCT constructs within the data collected. This approach can interpret rich data, synthesise various topics, and highlight relationships between several emerging themes [68]. Therefore, themes within the data capture important relationships between meanings inside the dataset and represent a level of importance within the overall topic. The thematic coding commenced with a deductive approach based on social cognitive theory (SCT) until saturation occurred. A secondary inductive approach was utilised for the emerging themes found outside the theoretical model. The additional codes were developed to capture emergent themes that reoccurred throughout each interview, whether these appeared linked to the theory or not. This proceeded until saturation was reached, and from there, a hierarchy of themes was developed. A total of 164 pages of transcripts were coded, separately, by two independent researchers to ensure the reliability and validity of the coding. Any differences were discussed and moderated. Below in Table 1 is an example of the hierarchy of coding and themes. The interviews were analysed as a whole set of interviews and then comparisons were made between the two populations afterwards to determine if there were any similarities or differences between the two.

## 3. Results

The participant sample comprised of 24 employees/personnel (n = 12 military personnel and n = 12 mining employees). There were similarities and differences between the two populations in terms of demographics. The military personnel had 83.4% males and 16.6% females, which broadly reflects the Australian Defence Force workforce that is comprised of 18% females [72]. The mining employees were 83.4% males and 16.6% females, which broadly reflects the general Australian mining workforce, which is comprised of 17% females [73].

The military personnel were younger (average age 21 years). They had been in the military for 1–3 years, while the mining group were older (average age 45) with 10+ years within the mining industry. The percentage of breakfast eaters to skippers were 25% in the military group and 17% in the mining group. All but two breakfast skippers were female participants.

### 3.1. Breakfast Eating Behaviours

The participants who ate breakfast as a child still ate now, and the participants who skipped breakfast during childhood were still skipping. When asked why they still ate the same foods, most participants acknowledged it was what they always ate, providing habitual behaviours. People who stated they ate breakfast described it as their first meal of the day and described eating what would be considered typical breakfast foods, both hot foods (such as eggs and bacon) and cold breakfasts (such as toast and cereal). A few participants mentioned delaying breakfast, although they still considered they were having breakfast, not simply “skipping” and having another meal later. Mining employees indicated they ate breakfast more often than military personnel. Military personnel described skipping breakfast at work most of the time, but would grab something fast and easy on the go at home, compared to mining employees who would prefer a sit-down meal each morning with their work colleagues. In most cases, participants described eating similar types of breakfast foods to the types they ate during their childhood. Three mining employees stated they have changed their habits due to energy balances (feeling dizzy) or needing to amend their eating behaviour for other reasons such as weight control. Military personnel described eating with their families at home more often than the mining participants. The military personnel in this study could leave the base and go home each night (but still had access to the food provided on base if they wished), whereas the mining employees were fly-in fly-out (FIFO) workers, meaning that their only options for meals were the dining services at the mining site.

### 3.2. Behavioural Influences

Participants demonstrated skills around cooking breakfast, cooking for themselves daily, and understanding how to cook different hot and cold breakfast meals. Their descriptions of the foods they ate for breakfast provided some evidence of a range of skills, and self-efficacy to perform a range of breakfast preparation behaviours and achieve consumption of breakfast when they wanted to. Participants often described that they would eat differently when at work compared with when they were at home, which suggests they had formed practices and routines that were more suited to each situation. They justified these differences as a response to time constraints, food availability, or low motivation to cook hot breakfasts at home. Military participants described that they often preferred to skip breakfast at home due to limited access to appliances, or they described making quicker and convenient breakfast meals like cereal. In general, self-efficacy appeared to be lower among military personnel, in that they were unable to prepare a nutritionally dense breakfast meal, or they were skipping breakfast altogether. Skills of understanding food packaging, nutritional quality, certain cooking skills, and preparation of foods were only demonstrated by having a general idea of certain food groups and healthy options as shown by this quote:

*I have a general idea. I would not say I knew exactly, but I do have a general idea [#11 mining].*

### 3.3. Cognitive Influences

Participants demonstrated knowledge of what is commonly called “breakfast” and what is generally considered a healthy breakfast through their descriptions of their past and current breakfast behaviours and the behaviours of others. Participants referred to what they thought to be a healthy breakfast, although healthy was interpreted in many different ways. For example, for some, healthy meant “lighter” (only having hot breakfasts occasionally), for others “protein” (to make a person feel full), and some had dietary needs that guided their choices at breakfast (gluten-free). Many participants shared a common expectation of breakfast—breakfast provided fuel for the day, energy, sustenance, and satiety to carry them through until the next meal. Without breakfast, participants expected to feel hungry or to be unable to push on through work. A few had modified aspects of their breakfast behaviours based on previous experiences, for example, feeling lethargic or tired if a big breakfast was eaten too early in the day. Participants could be described as having a positive or neutral attitude towards breakfast. These attitudes appeared to be related to the kinds of breakfast patterns they followed, in that those with a positive attitude generally followed more regular patterns involving a breakfast meal. In contrast, those who were neutral skipped breakfast from time to time and consumed convenient items (for example, toast). Mining employees had a more overall positive attitude towards breakfast eating and the expectation of what breakfast eating could provide for them throughout the day. Overall, mining workers understood the idea that breakfast can help them increase their energy and decrease hunger. Discussion around breakfast being the most important meal of the day was clearly evident for most mining workers, with their diet and weight control a significant factor described for eating breakfast. In general, institutional employees considered breakfast to positively impact their daily working life, as demonstrated by this quote:

*I think it is the energy, for sure. I feel I can attack the day much better if I have eaten breakfast than opposed to not eating breakfast. If I do not eat my breakfast and then I go out and start working, I generally feel sick from not eating, especially if I am starting to build it first thing in the morning. I think the food is a lot of fuel for the body [#1, mining].*

### 3.4. Environmental Influences

Workplace institutional settings are characterised by an “away-from-home” element that represents a change of environments for personnel or employees. Despite this, participants discussed varying degrees of social influences of family and/or friends on their breakfast eating behaviours. Most participants did not know what their friends or extended friends commonly did for breakfast, and only a few participants lived with immediate family, creating a situation where social influences are infrequently experienced. Some participants resisted social influence when their family was eating breakfast, stating that they skipped eating while others ate. Some participants who were parents provided the same breakfast options to their children, who ate exactly what they ate, demonstrating their social influence on their own children. Most participants did not realise they were setting the foundations for their children for their eating habits. Social eating on the mine site was mentioned by one participant who reported regularly eating breakfast with a group each morning.

A common theme from the interviews suggested that access to food options in a physical sense was not a barrier to breakfast eating, as both populations have the option of eating a breakfast meal that is provided each day. However, the workplace environment did create other barriers in the form of reduced time. Time was a significant barrier to breakfast consumption in both populations. Early morning personal training sessions (PT) were an issue for military personnel, who did not eat breakfast beforehand. Many military personnel reported they did not have time to eat at the mess after PT before their workday commenced. Military personnel advised that they would prefer to get extra sleep, or they would have something quick in their rooms (not necessarily a healthy breakfast), instead of going to get breakfast at the dining facility. Mine workers mentioned skipping breakfast on their days off, when sleep was more important to them. Military personnel also felt the limitations of their cooking appliances on base reduced their ability to prepare and eat breakfast. The distance to dining facilities for mining workers was a common example of why a participant would skip breakfast. An example of this is provided in the below quote:

*Because of the distance… I wake up, if you can save time for yourself and have more sleep, that is what I prefer [#10 mining].*

### 3.5. Other Social and Environment Factors Not Explicitly Described within SCT

Other environmental and social factors not described within SCT were related to institutional feeding services. Participants mentioned specific dietary practices, some based on advice from dietitians which is a social factor, some a result of dietary intolerances (such as intolerance to gluten) and some stemming from dietary preferences such as veganism. Despite the defence policy that contains mechanisms for individuals with special dietary requirements to request and receive meals to meet those requirements, participants felt the institutional feeding context did not offer alternatives suited to them, and they did not indicate an awareness of how they could make requests to access different options. This perception (of a lack of suitable options for those with particular dietary requirements) was also common in the mining sector. More commonly, negative perceptions of the feeding service were expressed. Claims were made from several participants in both populations on the quality of the cooking (under or over cooked), the nutritional quality of the food provided (fatty cuts of meat) and overall feel of the dining facilities (environment standards) were considerations (hit and miss) of why they didn’t attend those facilities. Participants from both populations advised that there was little to no guidance on how or what to eat in both workplaces as demonstrated by this quote:

*There is no guidance on that, and superiors have acknowledged that the standards not always the best, the qualities not the best. So, it is accepted as a very common to make your own food and arrange your own food [#3 military].*

There was no nutritional quality or ingredient list (environment factor) for each food item, and nor was there any indication of calories or serving sizes for the foods being provided. In particular, they felt they did not know enough about what they were eating. Participants from both populations indicated they would like to see further information in regard to healthy eating provided by their workplace, and they would like guidance on how to eat within their dining areas. Study participants asked for information to be provided in this area. In general, institutional personnel and employees considered standards and quality of the produce as negatively impacting their breakfast frequency at the dining facilities, as demonstrated by this quote:

*Generally, it is just the poor quality of foods. Mass produced stuff that is just not a very good standard [#1 military].*

## 4. Discussion

This study aimed to gather qualitative insights concerning breakfast eating by military personnel and mining workers whose lives are typified by away-from-home living on sites that provide institutional feeding services. Breakfast is important to ensure personnel can perform the physical and mental aspects of their workdays. Nine constructs from aocial cognitive theory were utilised to explore influences that could potentially cause the participants to skip breakfast. The analysis revealed additional social and environmental factors aligned within a social cognitive theory framework that can deliver an extended understanding of how rates of breakfast eating in workplace institutional settings can be increased. These will now be discussed in turn.

The cognitive aspects of breakfast consumption were the most prominent theme throughout the interviews. Participants discussed their thoughts and feelings on how breakfast either contributes to or derails the start of the day. Broader evidence also links cognitive thinking and control to breakfast consumption and improving cognitive control to keeping a healthy weight range [74,75,76]. Expectations, a cognitive thought underpinning the decision to eat breakfast, make the link between the food choice decision and any expected consequences of that decision. This can be related to taste, nutritional quality, social aspects, and cultural needs. Expectations of the outcome following eating breakfast are shaped by memories of past food, feelings and experiences from certain foods, or food consumption at certain times (i.e., breakfast). Similarly, recall of (positive) memories and feelings will enhance the enjoyment of specific timings and food preferences. Certain influences can also determine eating expectations [74,75,76]. This could be the expectation of taste, healthiness, or how the food is presented to influence a person’s food expectations. Within this study, most participants expectation of eating breakfast focussed heavily on the energy component of how it made them feel.

Environmental aspects can support or provide deterrents to breakfast eating. In this study, access, in terms of physical availability of foods did not appear to be an issue; however, a lack of time or the time it took to get to the dining facilities was discussed as an impediment. Both populations saw time as a barrier to breakfast eating, whether it was a distance to the dining facility, or having to perform physical training (which is mandatory for military personnel) prior to workday commencement, or simply they preferred to sleep in rather than get up early for breakfast before their long workdays. Studies have linked worse sleep scores and onset latency of sleep when skipping breakfast [77]; found people had more symptoms of anxiety and depression when skipping breakfast regularly [78,79], and breakfast skippers had less vigour and higher depressive scores than breakfast eaters [78,80,81]. Thus, being time-poor seems to increase the likelihood of skipping breakfast, which significantly impacts breakfast skippers both mentally and physically across several attributes [82]. In health promotion studies, impediments to access have been shown to contribute to breakfast skipping [83].

Social norms and the social influences on eating have been widely researched [75,84,85,86]; however, social norms and social influence were not described as influences on breakfast consumption for these two workplace institutional populations indicating participants are largely unaware of social influences. Again, this could be said for influence. There was only one participant acknowledging that he met up with work friends each morning for breakfast but did it out of habit as that was what they had always done. This participant was unaware of the social influence their group was having on each other to eat breakfast each morning. Influence on others, and whether family or friends eat breakfast, did not seem to cause these participants to either partake, or not, in breakfast overall.

Some of the SCT theoretical constructs explored directly in this research did not appear to have an influence on breakfast eating. Although SCT [59] proposes that all three triads play a role in behaviour, it may be the workplace institutional setting reduces the importance of some factors. In this study, the constructs of skills, practice, and self-efficacy (behavioural) were not seen to be determinants for breakfast frequency. Studies provide that self-efficacy has a strong correlation between eating behaviours and the ability for someone to feel confident in their food choices [87]. These constructs could be less important in an institutional setting, as personnel do not need food preparation skills and practice to prepare foods because breakfast is provided to them through a catering service. Social norms play a big part in food choice and behaviour, with social context having a powerful effect on people [75]. Research suggests that social norms are one of the most significant facilitators of what people eat, their sizing, and their health choices [88,89,90,91]. Within this study, the participants couldn’t report on what others were doing or eating around them and this may negatively affect their portion size and eating patterns.

While this study only asked specific breakfast related questions, it may be that the “come and go” nature of institutional workplaces may not be conducive to establishing the social bonds needed for social norms and social modelling to be effective. However, social modelling may have been a powerful influence earlier in the lives of these participants. The participants who ate breakfast as a child still eat breakfast now, and the participants who skipped breakfast during childhood are continuing to skip breakfast. This provided further evidence to the already considerable amount of research in this area [92,93,94], that habits and behaviours need to be changed and amended moving into adulthood, as they carry on to our adult years.

**Figure 1:**
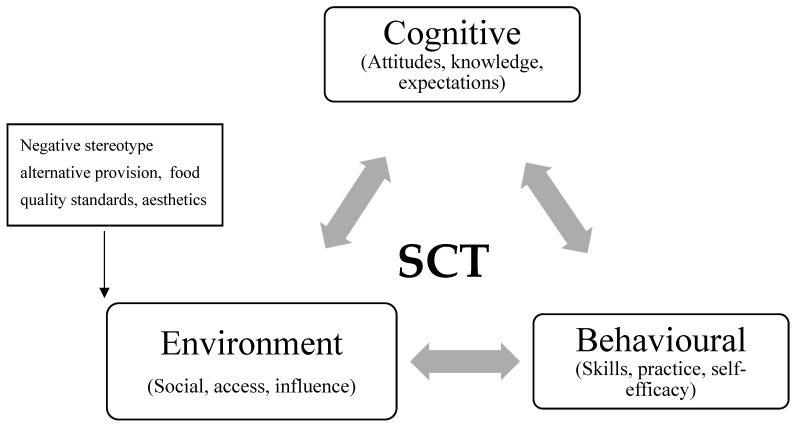
Initial and Emerging factors.

Interestingly, some social and environmental factors emerged that have not been described previously within the SCT framework. These can be seen as other influences created by workplace institutional settings that are not represented within the SCT reciprocal triad of interactions [59]. The initial and emerging social and environmental factors are shown below in Figure 1.

An emergent factor was the negative stereotype of institutional food services. Concerns about nutritional quality, taste, quality of the ingredients, and the method of cooking, and the level of available information to diners to allow them to understand what was in the foods provided were all described by participants in this study. This can be seen as both social and environmental factors that have not previously been conceptualised within SCT. These perceptions are attitudinal and are linked to expectations formed from past experiences with institutional feeding. This negative stereotyping of institutional food has been observed previously [95,96,97]. Poor satisfaction with dining facilities and expectations for food quality were again very apparent in this study. Attitudes and negative stereotyping of institutional food were discussed by participants from both populations as a factor in whether people ate breakfast. This is something to consider moving forward, as attitudes and expectations towards the behaviour (through the cognitive part of SCT) and towards the environment (in the environmental part of SCT) may need to change in order to change behaviour.

This research has provided further evidence that SCT is a valuable theory for use within healthy eating studies [87,88,89,90,91,98], with the influence of many theoretical constructs evident on breakfast consumption within institutional settings, with environmental and cognitive aspects most clearly described by participants in relation to breakfast frequency. One emerging theme, namely negative stereotyping of the workplace institutional food services, contributes to further development of the SCT theory. It may be necessary to extend considerations of the social and environmental influences on behaviour beyond the physical facilitators/barriers and the social influences, to include perceptions of social and environmental factors (in this case food services), which in turn determine whether individuals are drawn to that environment to take advantage of the opportunity to perform the behaviour within that environment. This indicates there is still work to be undertaken in developing the factors described within the environmental and social themes of SCT.

### Limitations and Future Directions

There were some limitations to this study. Firstly, the ratio of male and female participants was uneven. Even though the ratio was broadly reflective of both institutional settings, it may be advantageous to include more females in future studies to ensure their perspective is captured. Further limitations include the composition of the research team which was all female, and educated, which may have shaped the interview construction and the way participants responded within the interview process. This is acknowledged and future work should consider having gender differences in the research team. Furthermore, the interview questions were based on previously described SCT constructs, and this could be seen as narrowing the data capture. Given the timeframe of the research, the participants were not offered opportunities to comment on the accuracy of their interview information. In future studies, considerations should be made to invite participants to check and comment their transcriptions for accuracy of the data. Future work should include questions that probe other influences on breakfast frequency beyond SCT. Additionally, due to the focus of this study, and the nature of qualitative studies in general, no data was compiled about the health, performance, or workplace productivity of the participants to enable determination of whether breakfast consumption frequency was detrimental to those who skip breakfast. Due to the specific focus relating to workplace institutions, this work reflects perceptions of only two institutions cannot be generalised beyond this context.

Given the prominence of the cognitive elements of breakfast consumption, future research should focus on interventions that provide personnel and workers with information to increase their knowledge, improve their attitudes and create positive expectations for eating breakfast. Participants in both populations repeatedly mentioned that nutritional information would be highly beneficial to them, and nutrition related information or training should be initiated within these workplace institutions. Although both workplace institutions provide food services to ensure meals are available, strategies to improve the quality of the food, and the perceptions of the food services will assist in attracting personnel to the food services, which in turn increases the likelihood that they will consume breakfast more regularly. Given the need for both populations to eat healthier for work and performance, further research is warranted in this field through quantitative research to establish whether the influences on breakfast consumption identified here explain breakfast consumption across a broader sample of institutional workers. Research should focus on other ways to understand these barriers, and develop processes in the environment to help the workforce to overcome barriers faced.

## 5. Conclusions

Programs that aim to increase breakfast consumption in workplace institutional settings must create areas where their employees want to go. The negative stereotyping of institutional food services needs to be changed, food service provisioning environments need to ensure nutritious and high-quality food is produced. Employees would benefit from further information or education on healthy food choices and the importance of breakfast frequency, especially in young adults (military personnel), which could highlight the benefits of breakfast eating to establish positive expectations for regular breakfast consumption. Furthermore, timing plays a role in whether people eat breakfast, and a real or perceived lack of time reduces the ability of personnel and workers to eat breakfast, with many placing more importance on work commitments (physical training for military personnel) or sleep (both populations) than eating breakfast. In summary, consideration of SCT may assist program designers to build more effective interventions for institutional settings.

## Figures and Tables

**Table 1:** Initial coding hierarchy.

Parent Node	Child Nodes
Breakfast behaviours	Frequency, type, and ways of doing
Behavioural influences	Skills, practice, and self-efficacy
Cognitive influences	Knowledge, expectations, and attitudes,
Environmental influences	Social, access, and influence
New factors identified	food quality, service provision, alternatives provided, and dietary recommendations/guidance

## Appendix A

Interview Question Guide

Can you describe your first ever memory of eating breakfast? (cognitive)

Can you describe a typical breakfast scenario for your teenage years? (cognitive)

How has your breakfast eating changed from adolescence to now? (cognitive)

Did you eat breakfast today? (behavioural)

Tell me about it?

Probing questions

Yes—what was it, why did you choose this option today? etc.

No—why not? Constraints (behavioural/environmental)

If no, do others tell you that you should eat breakfast? What do they usually say? (Social)

Does your immediate family usually eat breakfast? Why? Why not? (social)

If yes, can you tell me a little about what they usually eat for breakfast? (social)

What do you expect to happen after eating breakfast? (cognitive)

Probing questions

Satiety, energy, etc.

Do you cook different breakfast options? (behavioural)

Do you know nutritional quality and quantity of most foods and labels?

Who taught you about breakfast food? (cognitive)

Do you feel confident to cook different breakfast foods? (behavioural)

How confident would you feel to cook new breakfast meals from a recipe? (behavioural)

When you eat out or at a Bayne Maree style food area, would you know how much food you are consuming? (behavioural)

Do you feel that you are confident to overcome obstacles to eat breakfast daily? (behavioural)

Do you feel your cooking skills are sufficient? (behavioural)

Can you prepare meals from scratch? (behavioural)

Do you enjoy planning and cooking meals? (behavioural)

Do you eat breakfast even if it decreases your daily sleep time? (environment)

Worksite questions

Did you eat breakfast the last day you were on the worksite? (Personal)

Probing questions

Yes—what was it, why did you choose that option? etc

No—why not? Constraints (Behavioural, Enviro)

Does your worksite encourage you to eat breakfast? What do they usually say? (Social)

Thinking about the last week. How many days did you eat breakfast? Why/why not?

When you are on the worksite, what kind of breakfast is provided each morning? (Environment) How are the breakfasts provided on the worksite different from home? (Environment)

Do your closest workmates usually eat breakfast? Why? Why Not? (Social)

Does your breakfast change daily? How does it change? Why doesn’t it change? (Environment)

What is the food like at work? (cognitive)

Do you like the food provided? (cognitive)

Is the food available to you at your work site? (Environment)

Do you have anything else to add about breakfast? (Personal)
